# Author Correction: FAN1 interaction with ubiquitylated PCNA alleviates replication stress and preserves genomic integrity independently of BRCA2

**DOI:** 10.1038/s41467-017-02130-x

**Published:** 2017-12-21

**Authors:** Antonio Porro, Matteo Berti, Julia Pizzolato, Serena Bologna, Svenja Kaden, Anja Saxer, Yue Ma, Kazuo Nagasawa, Alessandro A. Sartori, Josef Jiricny

**Affiliations:** 1 Institute of Molecular Cancer Research of the University of Zurich and ETH Zurich, Winterthurerstrasse 190, CH-8057 Zurich, Switzerland; 2 Department of Biotechnology and Life Science, Tokyo University of Agriculture and Technology, 2-24-16 Naka-cho, Koganei-shi, Tokyo, 184-8588 Japan; 3 Present Address: Wellcome Trust/Cancer Research UK Gurdon Institute, Tennis Court Road, Cambridge, CB2 1QN UK; 4 Present Address: Institute of Molecular Life Sciences of the University of Zurich and Institute of Biochemistry of the ETH Zurich, Otto-Stern-Weg 3, Zurich, 8093 Switzerland

*Nature Communications* **8**:1073 10.1038/s41467-017-01074-6; Article published online: 20 October 2017

The financial support for this Article was not fully acknowledged. The Acknowledgements should have included the following:

This study was in part supported by the Swiss National Foundation Grant No.: 31003A-156023 to Alessandro Sartori.

