# Supplementary material for: FAN1 interaction with ubiquitylated PCNA alleviates replication stress and preserves genomic integrity independently of BRCA2
Source: Nat Commun. 2017 Oct 20;8:1073. doi: 10.1038/s41467-017-01074-6 (PMC5648898; doi:10.1038/s41467-017-01074-6)
Supplement: Supplementary file 1 — Supplementary Information [file 41467_2017_1074_MOESM1_ESM.pdf]

FAN1 interaction with ubiquitylated PCNA alleviates replication stress and preserves genomic integrity independently of BRCA2

Antonio Porro[1]*, Matteo Berti[1], Julia Pizzolato[1], Serena Bologna[1†], Svenja Kaden[1], Anja Saxer[1], Yue Ma[2], Kazuo Nagasawa[2], Alessandro A. Sartori[1]* & Josef Jiricny[1¶]*

Supplementary Information

[1] Institute of Molecular Cancer Research of the University of Zurich and ETH Zurich, Winterthurerstrasse 190, CH-8057 Zurich, Switzerland. [2] Department of Biotechnology and Life Science, Tokyo University of Agriculture and Technology, 2-24-16 Naka-cho, Koganei-shi, Tokyo 184-8588, Japan. *Equally Contributing Authors. Present addresses: [†]Wellcome Trust/Cancer Research UK Gurdon Institute, Tennis Court Road, Cambridge CB2 1QN, United Kingdom; [¶]Institute of Molecular Life Sciences of the University of Zurich and Institute of Biochemistry of the ETH Zurich, Otto-Stern-Weg 3, 8093 Zurich, Switzerland.

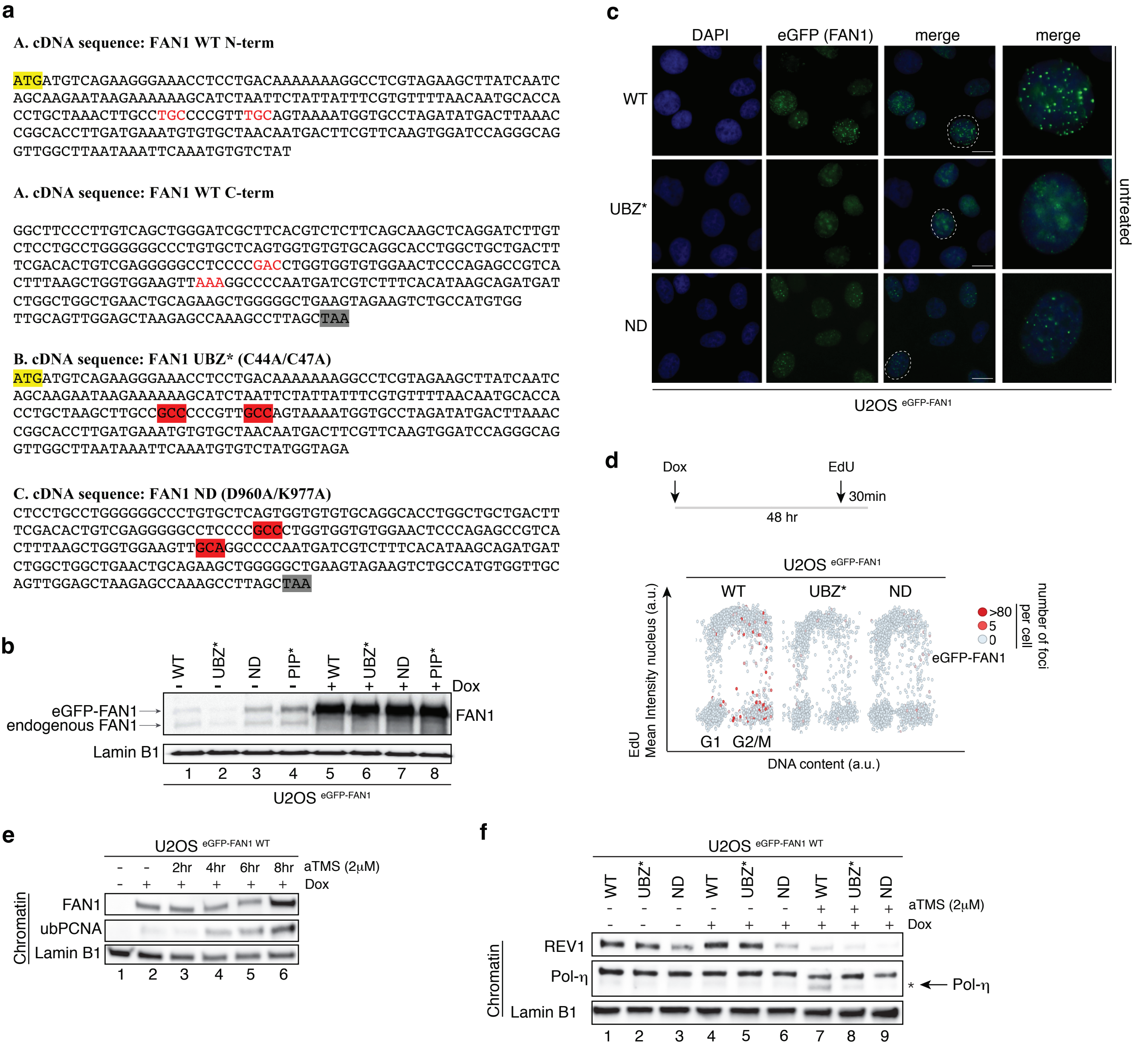

Supplementary Figure 1. Establishment and genotypic characterization of U2OS cells inducibly-expressing eGFP-tagged variants of FAN1. (a) Genomic DNA was extracted from U2OS cells inducibly-expressing the eGFP-tagged forms of FAN1 and subjected to PCR analysis with specific primers to amplify the coding sequence of ectopic FAN1. The resulting PCR products were sequenced to confirm the presence of the intended mutations in the UBZ and nuclease domains of the protein. **(b)** Immunoblot of total extracts of U2OS cells expressing the indicated eGFP-FAN1 variants. **(c)** eGFP FAN1 foci formation was evaluated in U2OS cells inducibly-expressing eGFP-tagged FAN1-WT (top row), FAN1-UBZ (UBZ*, second row) and FAN1-nuclease defective (ND, third row) mutants under unperturbed conditions. Representative images are shown. Scale bar: 25 μm. **(d)** QIBC of eGFP-FAN1 foci was performed in same cells as in b, pulse-labeled with EdU for 30 min. The heat map indicates the mean eGFP-FAN1 intensity per nucleus. **(e)** Chromatin-enriched fractions of U2OS cells inducibly-expressing eGFP-tagged full-length FAN1-WT were analysed by immunoblotting with the indicated antibodies 2, 4, 6 and 8 hr after aTMS (2 μM) treatment. **(f)** Chromatin-enriched fractions derived from U2OS cells inducibly-expressing eGFP-tagged full-length FAN1-WT, UBZ and ND mutants were analysed by immunoblotting with the indicated antibodies upon treatment with aTMS (2 μM; 24 hr). Asterisk indicates the band corresponding to Pol-η.

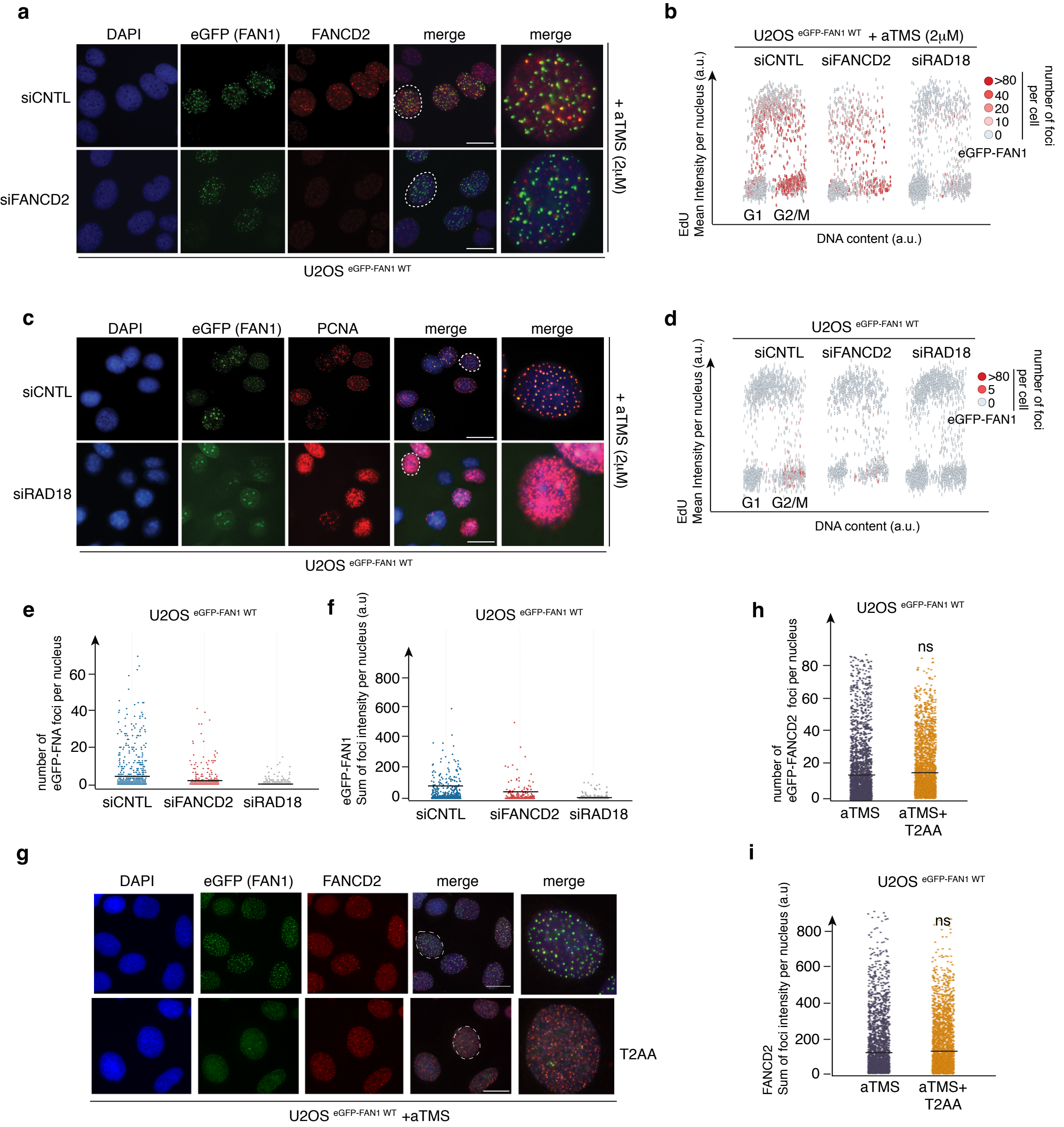

Supplementary Figure 2. FAN1 localization to aTMS-induced foci requires ub-PCNA but not FANCD2. **(a)** U2OS cells expressing eGFP-tagged FAN1 WT were transfected with the siRNA against FANCD2 and treated with aTMS (2 μM; 24 hr) before immunostaining with anti-FANCD2 antibody. Representative images are shown. Scale bar: 25 μm. **(b)** QIBC of eGFP-FAN1 foci was performed in same cells as in c transfected with the indicated siRNAs, exposed to aTMS (2 μM; 24 hr) and pulse labeled with EdU in the last 3 hr of aTMS treatment. The heat map indicates the mean eGFP-FAN1 intensity per nucleus. **(c)** U2OS cells expressing eGFP-tagged FAN1 WT were transfected with the siRNA against RAD18 and treated with aTMS (2 μM; 24 hr) before immunostaining with anti-PCNA antibody. Representative images are shown. Scale bar: 25 μm. **(d)** QIBC of eGFP-FAN1 foci was performed in U2OS cells expressing eGFP-tagged FAN1 WT transfected with the indicated siRNAs, left untreated and pulse-labelled with EdU for 30 min. The heat map indicates the mean eGFP-FAN1 intensity per nucleus. **(e, f)** Quantification of eGFP-FAN1foci count **(e)** and the sum of the intensities of eGFP-FAN1 foci **(f)** derived from the QIBC analysis in d. Median levels are indicated by black bars. **(g)** Immunostaining of eGFP-FAN1 and FANCD2 was performed in U2OS cells expressing eGFP-tagged FAN1 WT treated with aTMS (2μM; 24 hr) and incubated or not with T2AA (40 μM; 6 hr). **(h, i)** Quantification of FANCD2 foci count **(h)** and the sum of their intensities **(i)** was derived from the QIBC analysis of **g**. Median levels are indicated by black bars.

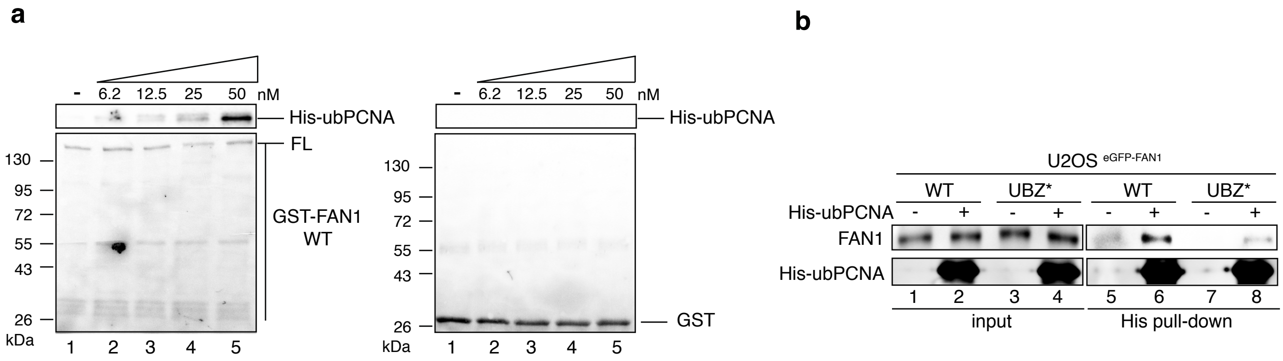

Supplementary Figure 3. FAN1 directly interacts with ub-PCNA through its UBZ domain in vitro and in vivo. (a) GST pull-down experiments were performed with a range of purified ub-PCNA concentrations using full-length GST-FAN1 WT or GST alone. ub-PCNA in pull-downs was analysed by immunoblotting. **(b)** Chromatin-enriched fractions derived from U2OS cells inducibly-expressing eGFP-tagged full-length FAN1-WT and the UBZ* mutant were incubated with purified His-ubiquitylated PCNA and subjected to His-tag pull-down experiments. The immuno-precipitates were analysed with antibodies against PCNA and FAN1.

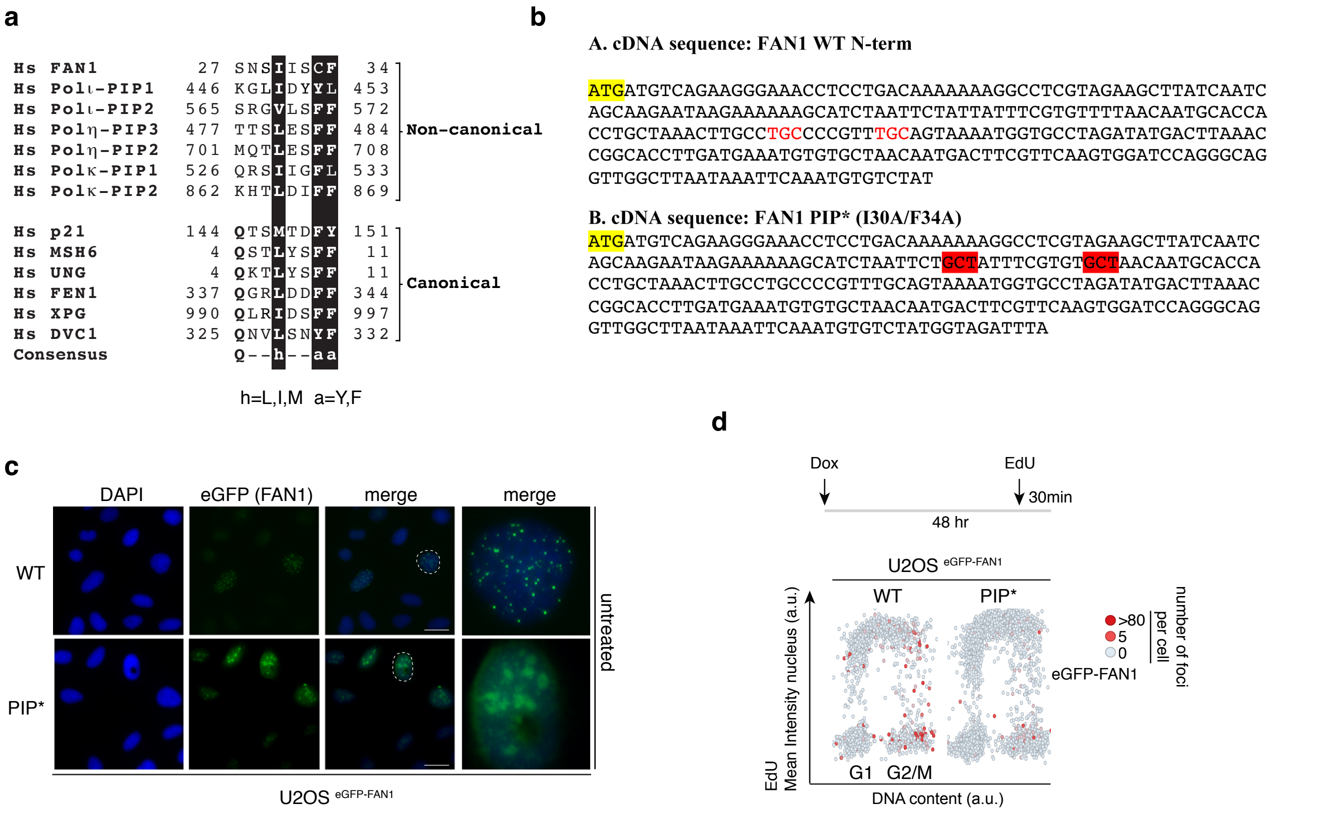

Supplementary Figure 4. Evolutionary conservation of the non-canonical FAN1 PIP-box motif and genotypic characterization of U2OS cells inducibly-expressing the eGFP-tagged FAN1 PIP* mutant. (a) The non-canonical FAN1 PIP-box motif was aligned with those of human Polι, Polη and Polκ (upper panel) or with the canonical PIP-box motif of human p21, MSH6, UNG, FEN1, XPG and DVC1 (lower panel). **(b)** Genomic DNA was extracted from U2OS cells inducibly-expressing the eGFP-tagged forms of FAN1 and subjected to PCR analysis with specific primers as mentioned above. The resulting PCR products were sequenced to evaluate mutations occurring in the PIP-box motif of the protein. **(c)** eGFP-FAN1 foci formation was evaluated in U2OS cells inducibly-expressing eGFP-tagged FAN1-WT and FAN1-PIP* mutant in unperturbed conditions. **(d)** QIBC of eGFP-FAN1 foci was performed in in unperturbed U2OS cells inducibly-expressing eGFP-tagged FAN1-WT and the FAN1-PIP* mutant pulse-labelledwith EdU for 30 min. The heat map indicates the mean eGFP-FAN1 intensity per nucleus.

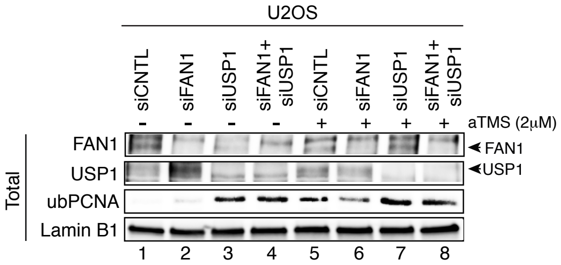

Supplementary Figure 5. Depletion of USP1 worsens genome instability in FAN1 depleted cells following aTMS treatment. Immunoblot of extracts of U2OS cells transfected with siRNAs against FAN1 and/or USP1, and treated or mock-treated with aTMS (2 μM; 48 hr). The antibodies used are shown on the left. A representative blot of three independent experiments is shown.

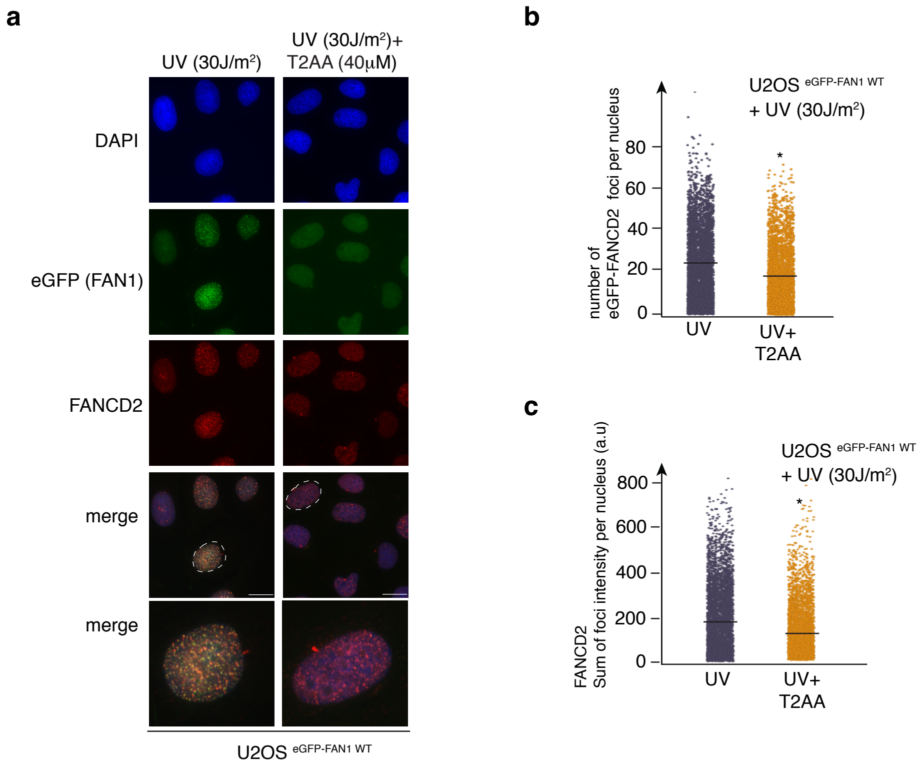

Supplementary Figure 6. The FAN1 interaction with ub-PCNA is required for FAN1 foci formation upon exposure to UV. (a) Immunostaining of eGFP-FAN1 and FANCD2 was performed in U2OS cells expressing eGFP-tagged FAN1 WT treated with UV (30J/m2; 4 hr release) and incubated or not with T2AA (40 µM; 6 hr). **(b, c)** Quantification of FANCD2 foci count **(b)** and the sum of their intensities **(c)** was derived from the QIBC analysis of **a.** Median levels are indicated by black bars. Statistical analysis was carried out using unpaired, two-tailed t-tests. *P* values expressed as * ($P<0.05$) were considered significant.

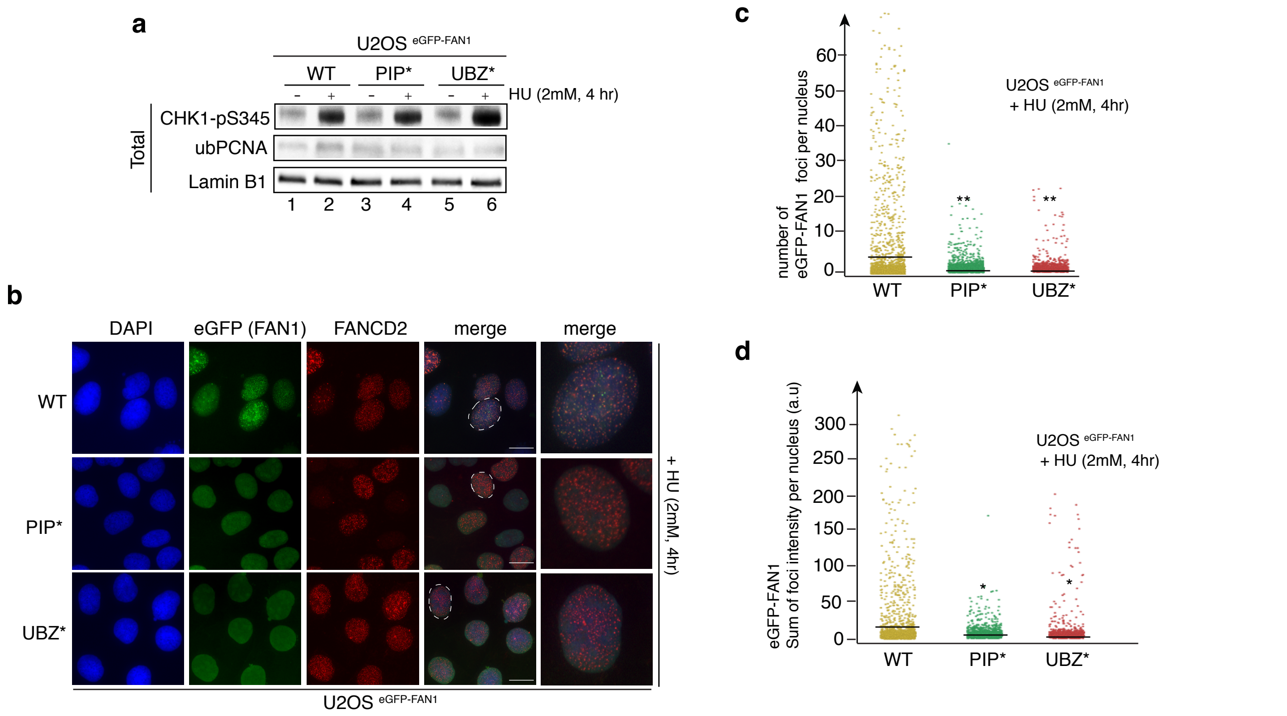

Supplementary Figure 7. The FAN1 PIP-box motif is required for FAN1 foci formation upon exposure to HU. (a) Immunoblot of total extracts derived from U2OS cells expressing eGFP-tagged FAN1 WT, PIP* and UBZ* and treated with HU (2mM; 4 hr). The antibodies used are shown on the left. Blots are representative of three independent experiments. **(b)** Immunostaining of eGFP-FAN1 and FANCD2 was performed in U2OS cells expressing eGFP-tagged FAN1 WT treated with HU (2mM; 4 hr) and incubated or not with T2AA (40 µM; 6 hr). **(c, d)** Quantification of eGFP-FAN1 foci count **(c)** and the sum of their intensities **(d)** was derived from the QIBC analysis of **b.** Median levels are indicated by black bars. Statistical analysis was carried out using unpaired, two-tailed t-tests. *P* values expressed as ** (P<0.01) or * (P<0.05) were considered significant.

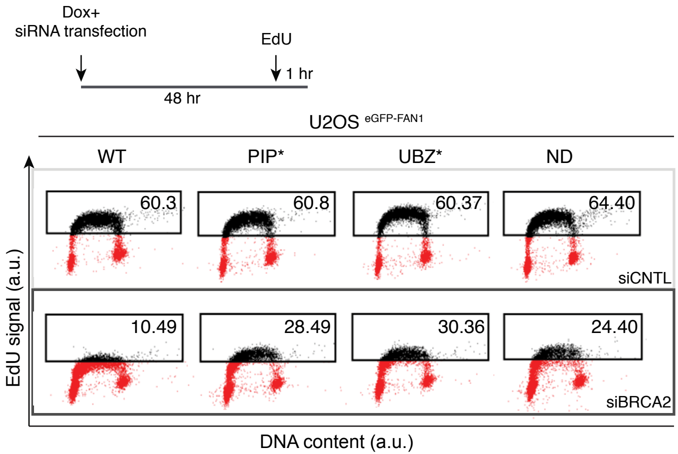

Supplementary Figure 8. The FAN1/ub-PCNA interaction limits DNA synthesis in BRCA2-deficient cells. U2OS cells expressing the indicated eGFP-FAN1 variants and depleted or not of BRCA2 were treated with EdU (1 hr) and Click chemistry 72 hr after the transfection with the indicated siRNAs . EdU incorporation was evaluated by FACS.

Figure 1a

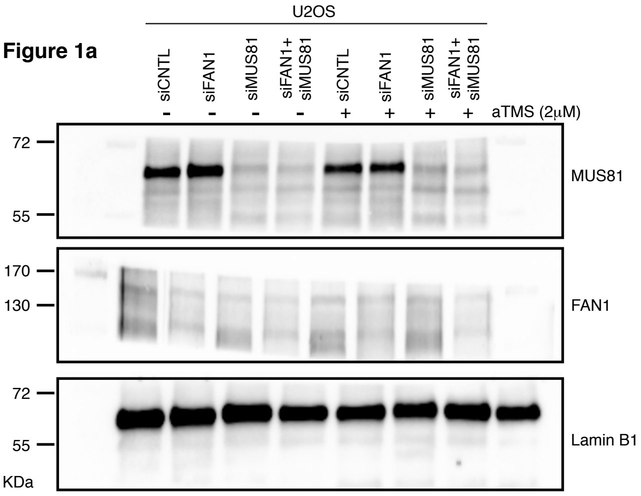

Figure 1h

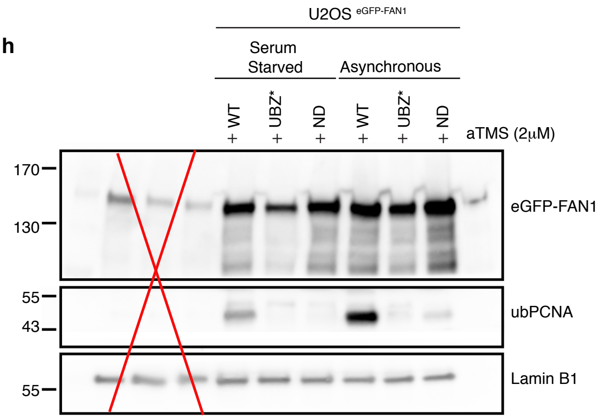

Figure 1g

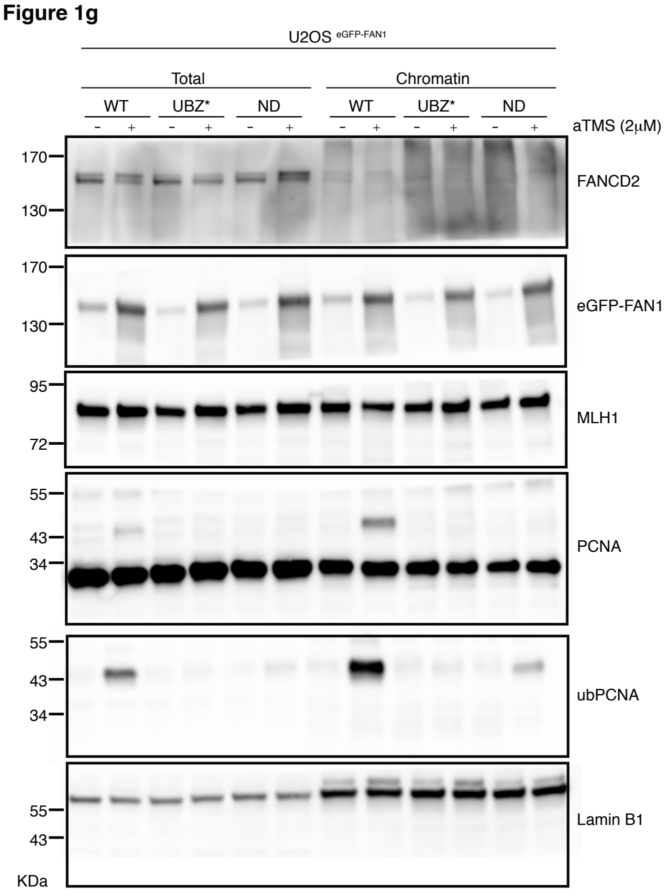

Figure 2a

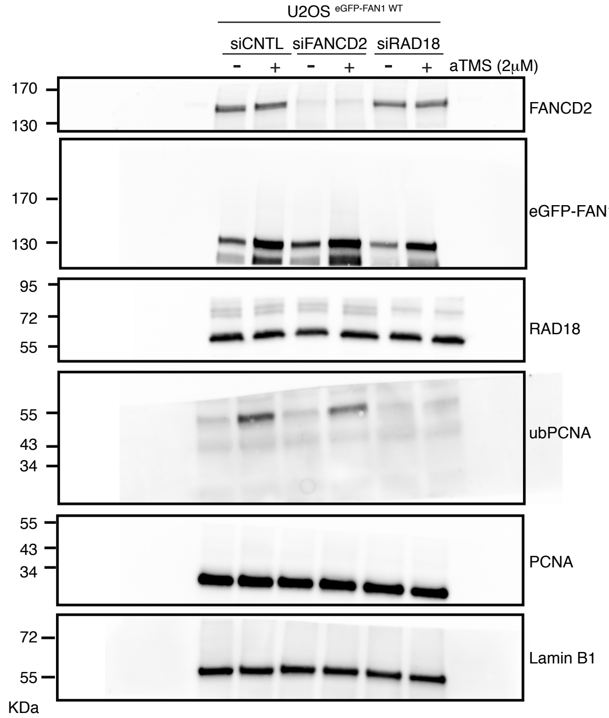

Figure 2e

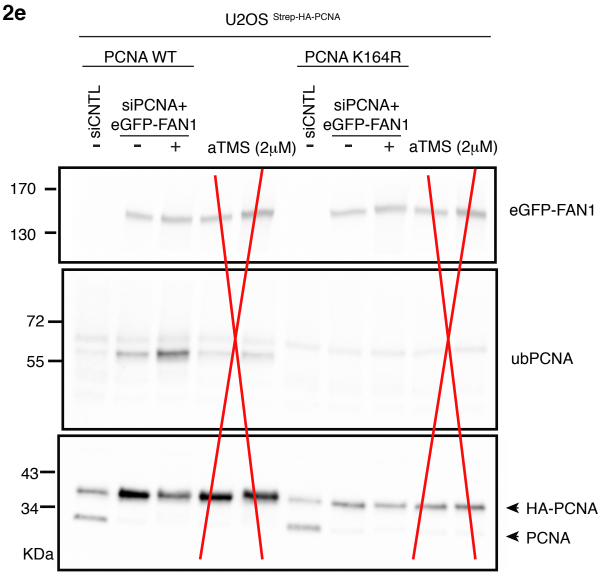

Figure 2g

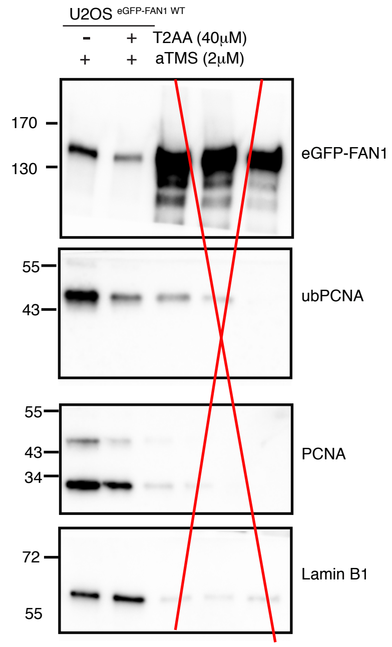

Supplementary Figure 9. Uncropped blots for Figure 1 and 2.

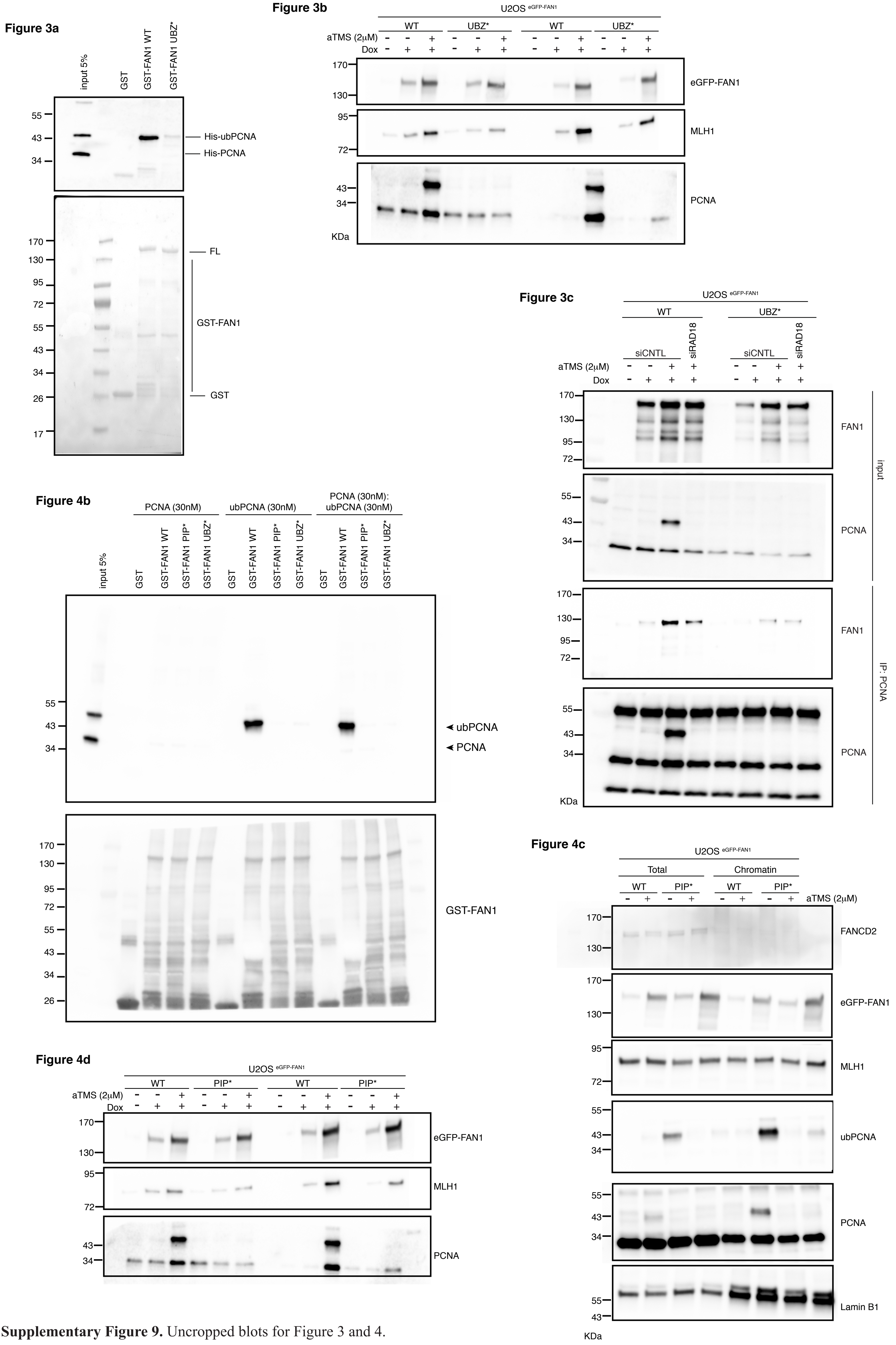

Supplementary Figure 9. Uncropped blots for Figure 3 and 4.

Figure 5a

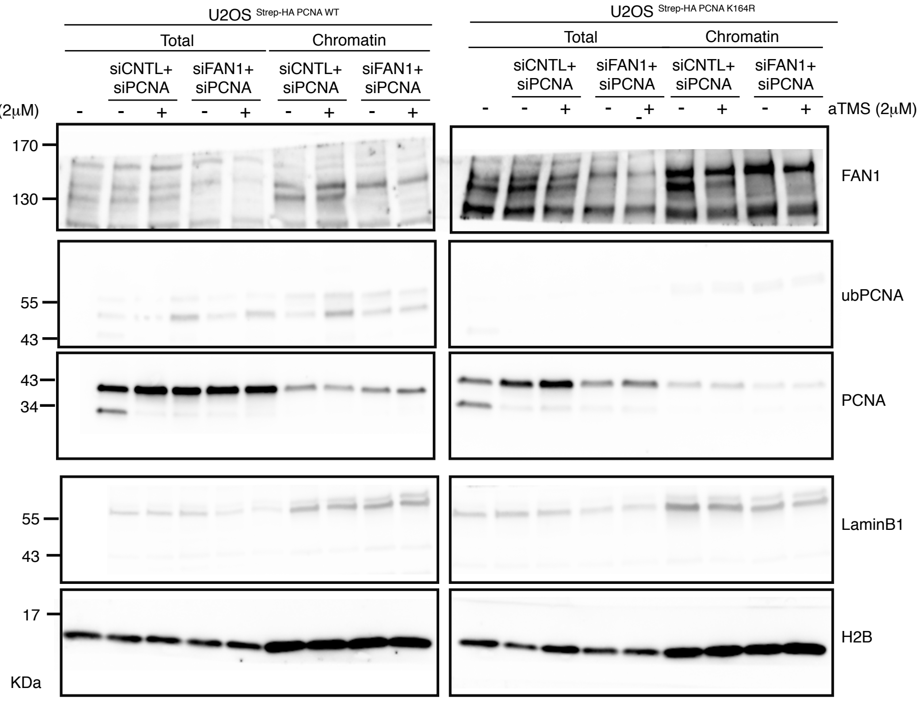

Figure 8a

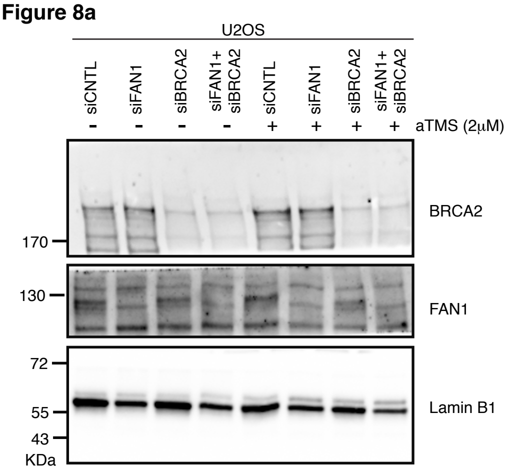

Figure 6i

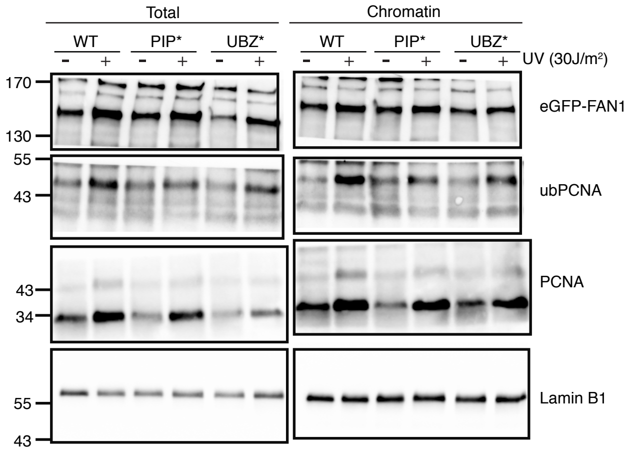

Figure 8f

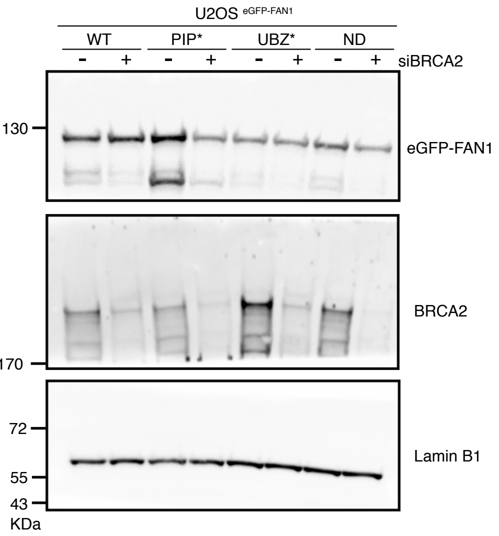

Figure 7a

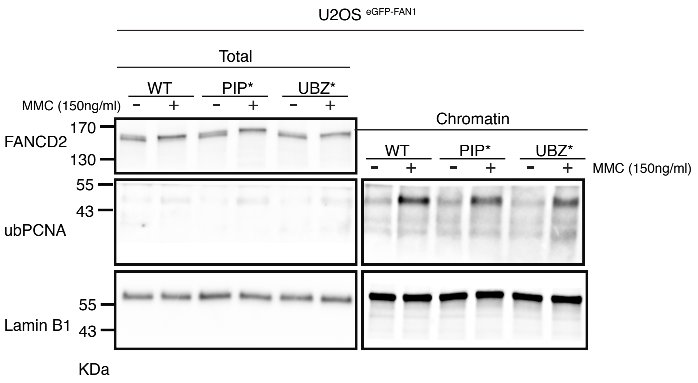

Figure 7e

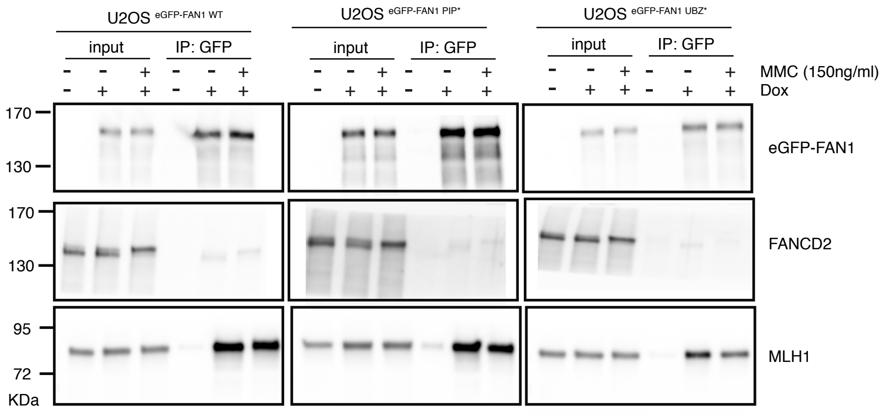

Supplementary Figure 9. Uncropped blots for Figure 5,6,7 and 8.
